# Case report: Prostatic malakoplakia: a rare disease that has a profile mimicking prostate cancer

**DOI:** 10.3389/fonc.2024.1348797

**Published:** 2024-04-11

**Authors:** Yelei Ren, Weihao Chen, Mengni Zhang, Xuhui Zhang, Jiaojiao Zhou, Yongzhong Li, Diming Cai

**Affiliations:** ^1^ Department of Medical Ultrasound, West China Hospital, Sichuan University, Chengdu, China; ^2^ West China School of Medicine, West China Hospital, Sichuan University, Chengdu, China; ^3^ Department of Pathology, West China Hospital, Sichuan University, Chengdu, China

**Keywords:** prostatic malakoplakia, prostate-specific antigen, ultrasound, magnetic resonance imaging, pulmonary disease

## Abstract

Prostatic malakoplakia (PMP) is a rare inflammatory disease, and misdiagnosis on imaging is a major reason for unnecessary punctures; however, information on imaging is even rarer. Five patients with PMP between May 2022 and February 2023 were enrolled in this study to summarize the imaging manifestations. All patients underwent ultrasound (US)-guided prostate biopsy and were confirmed by pathology, and the presence of prostate cancer was also excluded by pathology. The five patients, with a median age of 71 years (range = 58–74 years), had a median total prostate-specific antigen (T-PSA) of 10.40 ng/mL (range = 1.74–63.42 ng/mL). In two patients, chest computed tomography showed pulmonary infections. All patients underwent magnetic resonance imaging (MRI). Of these patients, four had a Prostate Imaging–Reporting and Data System (PIRADS) score of 5, while one had a score of 4. The lesions were mostly distributed in the peripheral zone of the prostate and appeared as a high signal on T1-weighted imaging (T1WI) and a low signal on T2-weighted imaging (T2WI). In the US examination, four patients had abnormal prostate morphology, with an unsmooth envelope and non-uniform parenchymal echogenicity. Four patients had increased prostate volume. US showed a hypoechoic nodule with non-uniform internal echogenicity, and an abundant internal blood flow signal was detected by color Doppler US. PSA, MRI, and US were not specific for PMP in our study, but we found that a history of co-infection may be helpful in an accurate diagnosis and to avoid unnecessary biopsy.

## 1 Introduction

Malakoplakia is a rare chronic inflammatory disorder that was first observed by Michaelis and Gutmann, who named it in 1902 (1). It is commonly found in immunocompromised patients and frequently invades the urinary system, particularly the bladder (2). Prostatic malakoplakia (PMP) is an extremely rare condition (3–5). It usually occurs in men over the age of 60 and may be overtly symptomatic or clinically silent. A puncture biopsy remains the gold standard for diagnosis (5, 6). Imaging information on PMP is extremely limited, which may cause misdiagnosis and unnecessary punctures. In this study, we retrospectively included patients who had confirmed PMP and excluded prostate cancer by pathology after prostate biopsy at our institution between May 2022 and February 2023 to further describe the clinical manifestations, pathology, laboratory tests, and imaging performance of this rare disease.

## 2 Case presentation

### 2.1 Clinical manifestations and laboratory test findings

This series included five patients with a median age of 71 years (range = 58–74 years), a median total prostate-specific antigen (T-PSA) of 10.40 ng/mL (range = 1.74–63.42 ng/mL, reference value <4ng/mL), a median free prostate-specific antigen (F-PSA) of 0.805 ng/mL (range = 0.135–2.92ng/mL, reference value <0.75 ng/mL), and a median free/total (F/T) ratio of 7.74% (range = 4.6%–16.57%, reference value 25%–100%). The results of the general physical examinations of the patients were normal. Four had elevated T-PSA levels, three had elevated F-PSA levels, and all had low F/T. Only one patient came to clinic due to frequent, urgent, and painful urination; the other patients were clinically silent (**Table 1**). The chest computed tomography of two of the patients indicated coexisting pulmonary infections. Quantitative urine sediment analysis was available for two patients, with one showing qualitative urine protein of 0.3 g/L (1+), nitrite (++), occult blood of 167 cells/μL (2+), white blood cell (WBC) count of 500 cells/μL (3+), urinary sediment bacteria of 12,271/μL (reference value < 230/μL), and electrical conductivity of 8 mS/cm (reference value = 19.8–42.5 mS/cm). Another patient had a WBC count of 15/μL in the urine sediment (reference value = 0–11/μL) and an electrical conductivity of 13 mS/cm. The urine culture was available for one patient, with the isolated bacteria including *Escherichia coli*. No other malignant tumors were found in any of the patients.

**Table 1:** Clinical data and ultrasound presentation of five patients with prostatic malakoplakia.

			1^a^	2	3	4	5
Age (years)			58	72	68	71	74
Lower urinary tract symptoms			Absent	Absent	Absent	Present	Absent
Comorbid symptoms/diseases			–	–	Pulmonary infections	Pulmonary infections	Hypertension
					Hydrocele of tunica vaginalis		Abnormal pelvic lymph node morphology
T-PSA (ng/mL)			10.400	8.680	63.420	1.740	16.900
F-PSA (ng/mL)			0.805	0.521	2.920	0.135	2.800
Ultrasonography							
Prostate	Volume^b^ (mL)		57.63	58.71	55.68	28.85	144.97
	Morphology		Slightly abnormal	Slightly abnormal	Abnormal	Normal	Abnormal
	Envelope		Slightly unsmooth	Slightly unsmooth	Unsmooth	Smooth	Unsmooth
	Echoes		Slightly non-uniform	Slightly non-uniform	Non-uniform	Uniform	Non-uniform
	Nodule		Absent	Absent	Present	Absent	Absent
Nodules	Location		–	–	Peripheral zone	–	–
	Size				51×11mm		
	Boundary				Unclear		
	Echoes				Non-uniform		
	Internal blood flow				Increasing		
Treatments			Follow-up observation	Follow-up observation	Transurethral electrolysis of the prostate	Transurethral electrolysis of the prostate	Transurethral electrolysis of the prostate

T-PSA, total prostate-specific antigen; F-PSA, free prostate-specific antigen.

a Numbered according to the patient’s arrival time.

b Volume was calculated as: (left and right diameter × anterior and posterior diameter × upper and lower diameter) × 0.52.

### 2.2 Multi-parameter magnetic resonance imaging findings

All MRI examinations were performed on a 3.0-T MRI system (uMR 780, United Imaging Healthcare, Shanghai, China) with a phased-array body surface coil. A multi-parameter prostate magnetic resonance imaging (MP-MRI) protocol, which included T1-weighted imaging (T1WI), T2-weighted imaging (T2WI), micro-view diffusion-weighted imaging (DWI), and three-phase dynamic contrast-enhanced (DCE) imaging using a three-dimensional (3D) fat-suppressed spoiled gradient-echo T1W sequence, was acquired. DWI with three different *b*-values (50, 200, and 1,400 s/mm^2^) was obtained, and apparent diffusion coefficient (ADC) maps were calculated and constructed based on two *b-*values (1,400 and 50 s/mm^2^).

**Figure 1:**
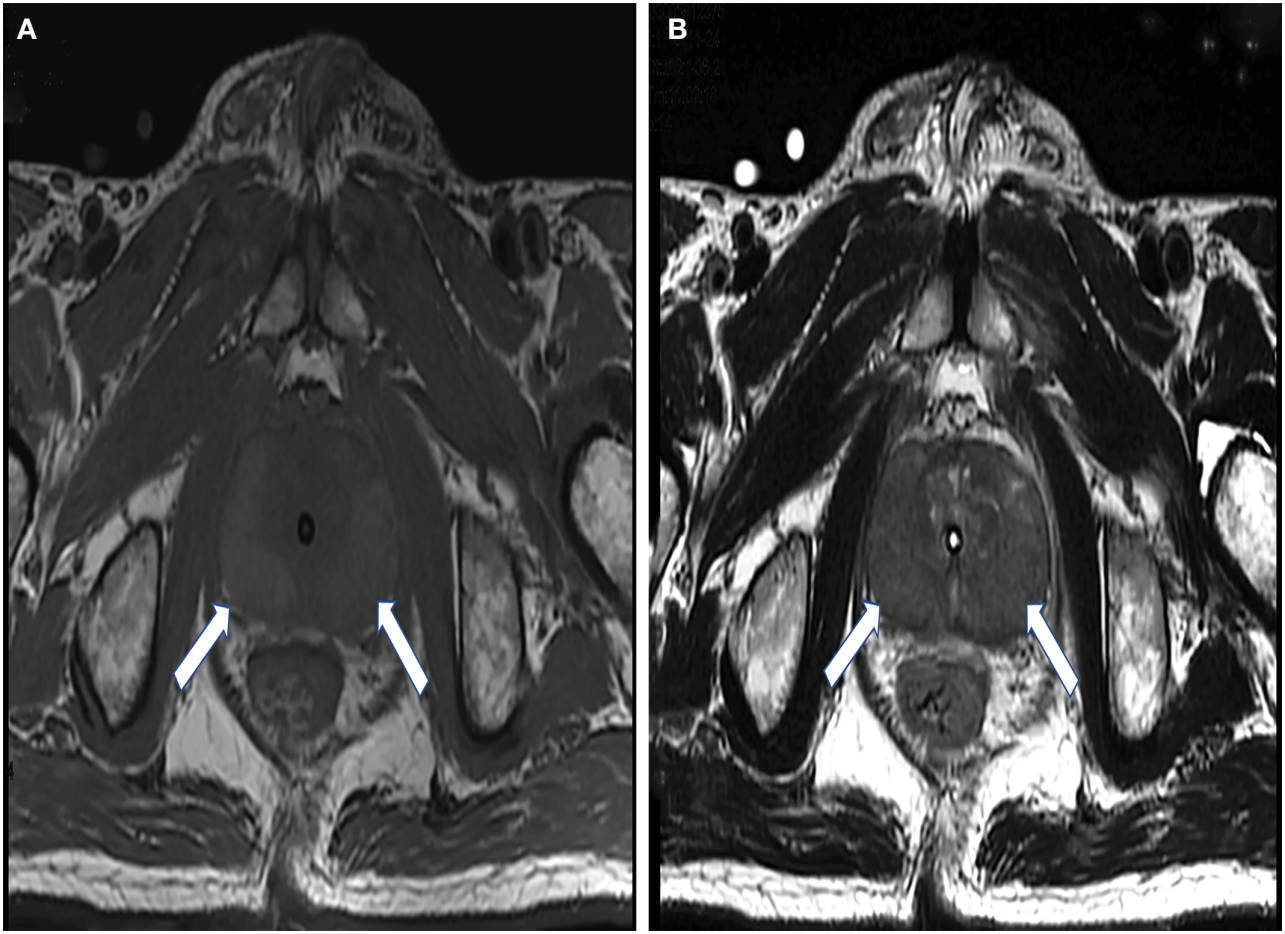
A 68-year-old man with elevated prostate-specific antigen (PSA). The lesions (*arrows*) showed a high signal on T1-weighted images **(A)** and a low signal on T2-weighted images **(B)**.

MP-MRI was available for all patients. Four of the patients had a Prostate Imaging–Reporting and Data System (PIRADS) score of 5, while another had a score of 4. The results showed bilateral involvement of the prostate, and the lesions could be located in the peripheral zone, central zone, and migratory zone. Peripheral zone involvement was the most common. The lesions mostly showed a high signal on T1WI, a low signal on T2WI (**Figure 1**), a high signal on DWI, and a low signal on ADC. A more uniform and obvious strengthening after enhancement was also seen. One patient showed abnormal lymph node morphology in the pelvis and suspected tumor metastasis or infection.

### 2.3 Ultrasound examinations

**Figure 2:**
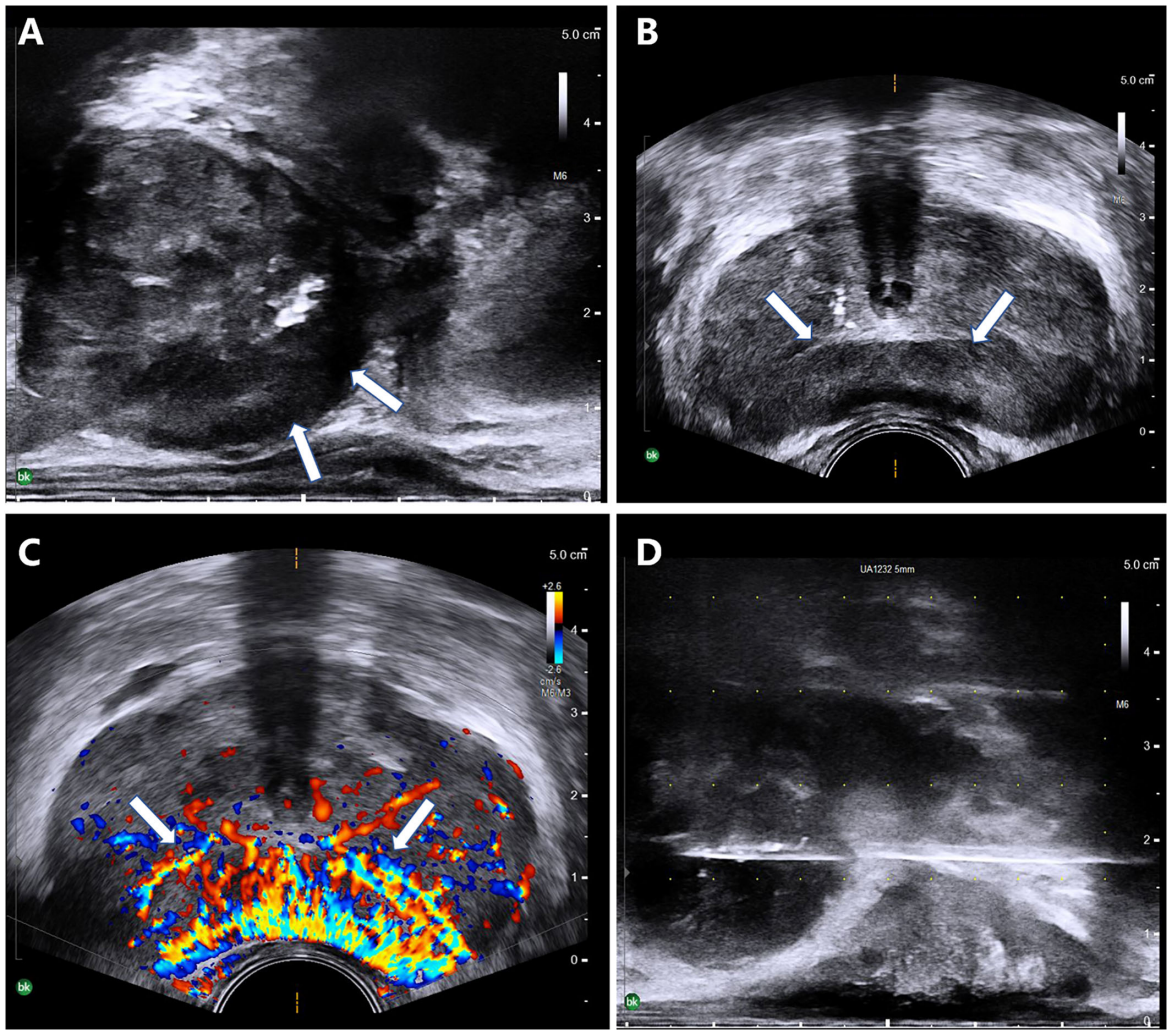
A 68-year-old man with elevated prostate-specific antigen (PSA). **(A–D)** Ultrasound revealed a morphologically abnormal prostate **(A)** and a hypoechoic nodule (*arrows*) at 3–8 o’clock in the peripheral zone **(B)**, with an abundant blood flow signal in it **(C)**. **(D)** A transperineal prostate biopsy was performed under ultrasound guidance.

All of the patients underwent ultrasound (US) examinations and a prostate biopsy with US guidance. The transperineal approach was chosen. The US images showed four of the patients having abnormal prostate morphology, with an unsmooth envelope and non-uniform parenchymal echogenicity. Four patients had increased prostate volume (**Table 1**). Only one nodule was detected by US, the size of which was approximately 51 × 11 mm, at the 3–8 o’clock direction in the peripheral zone. The boundary of the lesion was not clear, and the internal echogenicity was not uniform. The internal blood flow signal increased significantly. In addition, a strongly echogenic calcified focus in the prostate parenchyma was detected by grayscale (**Figure 2**).

### 2.4 Pathological findings

**Figure 3:**
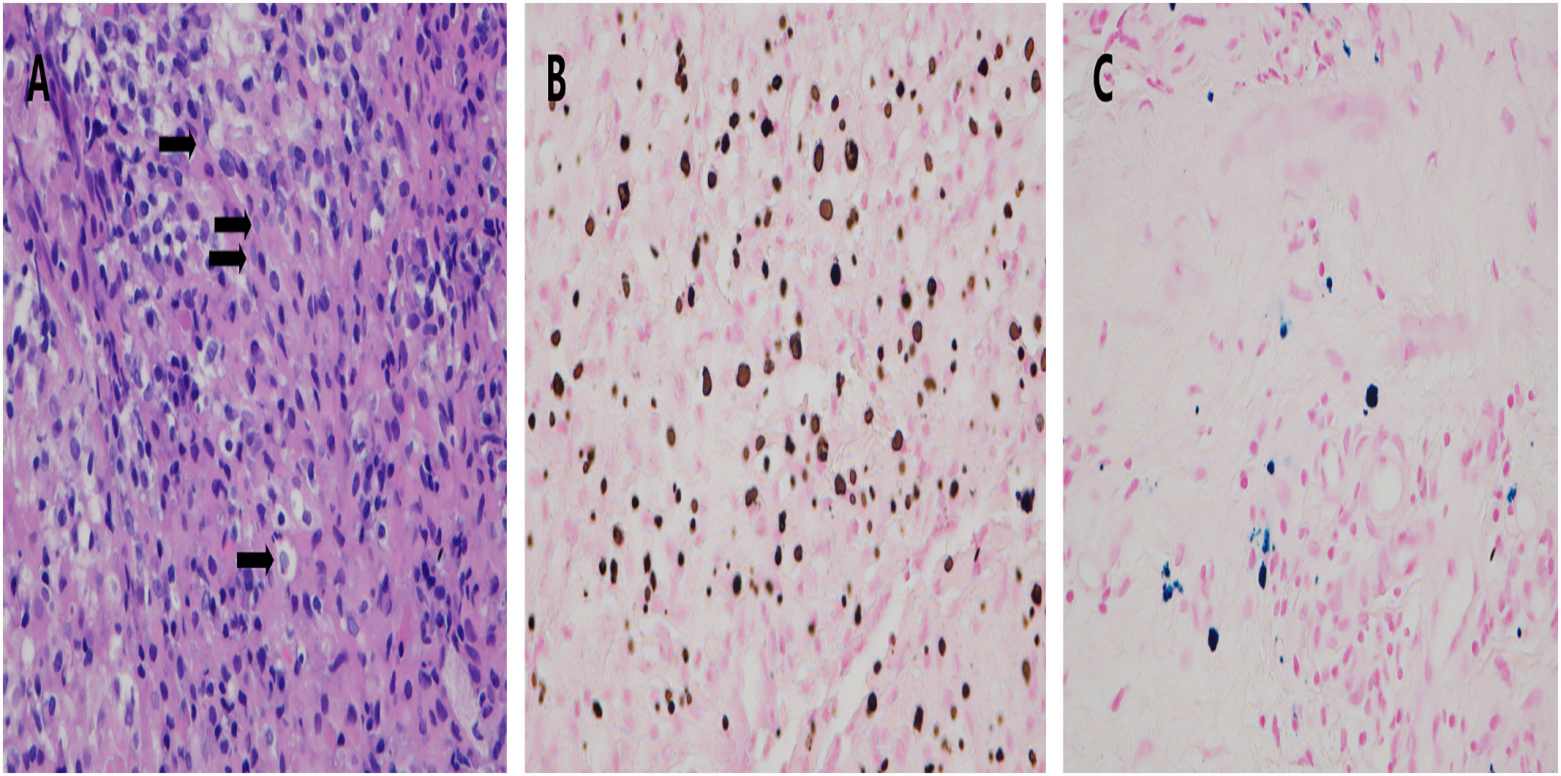
Prostatic malakoplakia misdiagnosed as prostate cancer. A large number of Michaelis–Gutmann bodies (*arrows*) were seen in the interstitium. **(A)** Histochemical staining showed large intracellular deposits of calcium **(B)** and iron **(C)**.

All patients underwent pathological examinations, with the results indicating PMP. All of the patients showed a large number of histiocytic infiltrates in the interstitium, with lymphocytic infiltration seen in two patients, eosinophilic infiltration seen in one patient, and focal granulomas seen in one patient. Histochemical stains showed calcium staining of Michealis–Gutmann (MG) bodies (+), PAS (+), iron staining (±), and antacid (−) (**Figure 3**). In the patients for whom prostate adenocarcinoma could not be directly excluded, further immunohistochemistry was performed. Three patients showed CD68 (histiocyte +), and two patients showed P63 (+) and high-molecular-weight cytokeratin (HCK) (+) in immunohistochemistry. PCR for *Mycobacterium tuberculosis* (TB-PCR) was performed in four patients, but no *M. tuberculosis* DNA fragments were detected.

## 3 Discussion

The pathogenesis of malakoplakia is still not clear. It frequently occurs in immunocompromised patients (7), and the impairment in the bactericidal activity of the macrophages could be important in its pathogenesis (8). Malakoplakia has a different prognosis depending on where it occurs. Studies have shown that conservative medical intervention through the use of antibiotics may be effective, but surgical intervention may sometimes be necessary (6, 9–12). There are only isolated reports on malakoplakia imaging in the literature, which recognize that the imaging findings of malakoplakia often mimic different diseases and cancers. PMP is extremely rare, but imaging information on it is even rarer (13, 14).

PMP can be easily misdiagnosed as prostate cancer due to similarities in the clinical symptoms and laboratory findings (15). Both have a similar peak incidence period over 60 years of age and similar clinical symptoms, including lower urinary tract infection or urinary tract obstruction. Moreover, they have elevated PSA on serological testing (4, 16–18). On MP-MRI, both show similar presentations, with the lesions being commonly found in the peripheral zone of the prostate and showing a low signal on T2WI, a high signal on DWI, a low signal on ADC, and an early enhancement with retention of the contrast on DCE (19, 20). In this study, most patients (4/5, 80%) did not present with significant clinical symptoms, and the PSA level of one patient (1/5, 20%) was normal. All of the patients had a PIRADS score of 4 or 5, and one patient showed abnormal pelvic lymph node morphology, which caused high suspicion of prostate cancer. These demonstrate that the clinical features of PMP are not unique and that it is difficult to correctly distinguish PMP from prostate cancer. Pathological findings provide strong evidence for the diagnosis of PMP. The histological features of PMP are macrophages containing calcified lysosomes, called MG bodies, which is the main factor that could differentiate PMP from prostate cancer (21, 22). However, finding a noninvasive and effective imaging tool for the diagnosis of PMP can avoid unnecessary punctures.

US is widely used in the evaluation of prostate disease because of its convenience and inexpensive features (23). The transrectal approach to US of the prostate is the method of choice; however, transabdominal approaches are also available when the patient does not meet the conditions for the transrectal approach (24). The literature indicated that the US of PMP could depict localized hypoechoic areas with indistinct borders and uneven internal echogenicity in the prostate (15, 22, 25). In this study, four patients had an elevated PSA level, and their US showed the prostate to have an abnormal morphology, an unsmooth envelope, and non-uniform internal echogenicity. A hypoechoic nodule with irregular morphology and non-uniform internal echogenicity was detected by the gray-scale mode, while color Doppler US showed an abundant blood flow signal within it. These results were similar to those reported in a previous study (18). These presentations of gray-scale US and color Doppler US are similar to those of prostate cancer. In this study, US could not differentiate PMP or prostate cancer, including MP-MRI (26).

Some studies are of the opinion that defective bacterial phagocytosis and lysosome function are the cause of the disease, as most cases also present with bacterial infections, 80% of which were *E. coli* (2, 9). Previous studies have also reported two patients with malakoplakia with bronchial asthma and another three with pulmonary cancer (10, 27). In this study, two patients (2/5, 40%) had pulmonary infections, which implies that the occurrence of PMP may be related to pulmonary disease. Combining the patient’s history of infection, pulmonary disease, and imaging findings might provide a correct diagnosis of PMP.

In recent years, some studies have shown that shear wave elastography (SWE) can be effective in the identification of prostate disease (28, 29). PMP is an inflammatory lesion in nature, and the prostate cancer tissue is generally harder than normal tissues. SWE can measure tissue stiffness and therefore could provide more significant information on PMP and be beneficial to avoiding unnecessary biopsies (30). However, further studies are needed.

This is a retrospective study. In addition, the number of cases collected was small due to the rarity of PMP. Insufficient comprehensive clinical data also resulted in an inability to discuss more details.

## 4 Conclusion

In conclusion, PMP is an extremely rare condition, and imaging reports on it are limited. The similarities between PMP and prostate cancer make diagnosis difficult. Pathological findings are still the most useful evidence to diagnose PMP. The occurrence of an infection in the body could be a signal, such as a pulmonary infection. For this disease, the combination of clinical manifestations and comorbidities could reduce the misdiagnosis rate.

## Data availability statement

The original contributions presented in the study are included in the article/supplementary material. Further inquiries can be directed to the corresponding author.

## Ethics statement

This study involving humans was approved by West China Hospital, Sichuan University. The studies were conducted in accordance with local legislation and institutional requirements. The participants provided their written informed consent to participate in this study. Written informed consent was obtained from the individual(s) for the publication of any potentially identifiable images or data included in this article.

## Author contributions

YR: Writing – original draft, Data curation, Formal analysis, Investigation. WC: Data curation, Investigation, Writing – original draft. MZ: Data curation, Formal analysis, Investigation, Writing – review & editing. XZ: Data curation, Investigation, Software, Writing – original draft. JZ: Data curation, Investigation, Writing – review & editing. YL: Investigation, Writing – review & editing. DC: Data curation, Investigation, Writing – review & editing.
